# A Rechargeable Biomineral Induced by the Sulfate-reducing Bacterium *Nitratidesulfovibrio* sp. HK-II

**DOI:** 10.1264/jsme2.ME24022

**Published:** 2025-07-03

**Authors:** Yui Arashi, Hiroki Mochihara, Hiroko Kubota, Kei Suzuki, Yusuke Chiba, Yutaka Kato, Toshihiro Kogure, Ryota Moriuchi, Hideo Dohra, Yuto Nakamura, Yosuke Tashiro, Hiroyuki Futamata

**Affiliations:** 1 Department of Applied Chemistry and Biochemical Engineering, Graduate School of Engineering, Shizuoka University, Hamamatsu, 432–8011, Japan; 2 Graduate School of Science and Technology, Shizuoka University, Hamamatsu, 432–8011, Japan; 3 Department of Earth and Planetary Science, Graduate School of Science, The University of Tokyo, 113–8564, Japan; 4 Research Institute of Green Science and Technology, Shizuoka University, Shizuoka 422–8529, Japan

**Keywords:** biogenic mineral, mackinawite, sulfate-reducing bacterium, microbial fuel cells, microbial ecosystems

## Abstract

A sulfate-reducing bacterium was isolated from the anode surface of a microbial fuel cell (MFC) producing a high current density. 16S rRNA gene ana­lyses showed that the isolate was affiliated with the genus *Nitratidesulfovibrio*, and the strain was named HK-II. When *Nitratidesulfovibrio* sp. strain HK-II was incubated anaerobically under sulfate-reducing conditions with Fe(III) citrate, a black precipitate formed. The resulting black precipitate was investigated using multidisciplinary methods. An X-ray diffraction (XRD) ana­lysis revealed that the black precipitate was mainly composed of mackinawite. A cyclic voltammetry ana­lysis showed clear redox peaks, and biogenic mackinawite possessed rechargeable properties. The XRD ana­lysis also showed that the form of the rechargeable biogenic mineral induced by strain HK-II (RBM-II) was changed by discharge and recharge treatments. Field-emission transmission electron microscopy revealed that lepidocrocite and amorphous iron oxide formed from mackinawite under discharged conditions, and the three mineral types were intermingled via charge and discharge cycles. Physicochemical parameters regularly changed under the treatments, suggesting that discharge occurred via iron oxidation followed by sulfur reduction and *vice versa*. These results indicate that sulfur dynamics are important key processes in charge and discharge mechanisms. MFCs equipped with lactate, strain HK-II, and an anode containing RBM-II consumed lactate under open-circuit conditions, after which MFCs generated a higher current density under reclosed-circuit conditions. These results demonstrate that RBM-II is a rechargeable material that enables the capture of electrons produced by bacterial cells and is useful for enhancing the performance of MFCs.

As a novel biotechnology using overlooked microbial functions, microbial fuel cells (MFCs) have been reported to generate electricity via the degradation of organic matter (Rabaey and Verstraete, 2005; Logan *et al.*, 2006; Lovley, 2006b; Rabaey *et al.*, 2007; Watanabe, 2008). Many researchers have attempted to increase current densities and decrease internal resistance, by optimizing structural designs (Tender *et al.*, 2002; Liu and Logan, 2004; He *et al.*, 2005), electrodes (Rubaba *et al.*, 2013; Suzuki *et al.*, 2016), and anode potential effects on current production (Torres *et al.*, 2009; Commault *et al.*, 2013; Suzuki *et al.*, 2018). However, despite improvements in MFC power density, modern MFCs as energy-producing devices still face limitations due to unresolved challenges in achieving higher current density. Conversely, MFC studies have simultaneously opened new frontiers, such as electroactive bacteria (Lovley, 2006a; Hau and Gralnick, 2007; Reguera and Kashefi, 2019; Ikeda *et al.*, 2021), microbial communities in MFC anodes (Lovley, 2012; Kato, 2015), and microbial extracellular electron transfer (EET) (Jung and Regan, 2011; Okamoto *et al.*, 2013; Ishii *et al.*, 2014; Shi *et al.*, 2016). These studies revealed electroactive bacterial diversity and the mechanisms underlying EET, leading to their application to wastewater treatment (Ishii *et al.*, 2013). In addition, novel relationships between microbes and minerals have been reported, providing insights into overlooked microbial survival strategies. Some bacteria produce the conductive nanoparticle FeS, consisting of pyrrhotite (Fe_1–x_S), mackinawite (Fe_1+x_S), and marcasite (FeS_2_), which localize extracellularly, intracellularly, and on the cell surface and exhibit efficient extracellular electron uptake (EEU) (Deng *et al.*, 2020) and EET (Kondo *et al.*, 2015) functionality. For example, *Shewanella loihica* strain PV-4 produces an iron monosulfide, and the assemblage of cells and mature iron monosulfide (mackinawite [Fe_1+x_S]) exhibits conductivity (Nakamura *et al.*, 2010; Kondo *et al.*, 2015). These findings have contributed to a more detailed understanding of previously overlooked microbial metabolism and will facilitate the development of new MFCs.

We previously reported that a MFC produced a significantly high current density for ~1 month (Suzuki *et al.*, 2018). Bacteria isolated from the anode surface of the MFC were analyzed in the present study to further investigate the high performance of the MFC. The isolated bacterium, *Nitratidesulfovibrio* sp. strain HK-II, induced the formation of a black precipitate in the presence of sulfate and ferric iron under anaerobic conditions. This black precipitate had a mineral-like appearance and was predicted to have conductivity, resulting in a high current density, similar to other conductive minerals (Nakamura *et al.*, 2010; Kondo *et al.*, 2015). We hypothesized that the black precipitate may be rechargeable, which has not been previously reported. If the black precipitate is a rechargeable material, it will provide support for the further development of MFCs. Therefore, the aims of the present study were to characterize the black precipitate using material science and electrochemical techniques, and to investigate whether it is valuable for increasing the efficiency of MFCs. We also discussed its rechargeable mechanism and geomicrobiological roles.

## Materials and Methods

### Isolation, incubation, and identification of bacteria

**Fig. 1.**
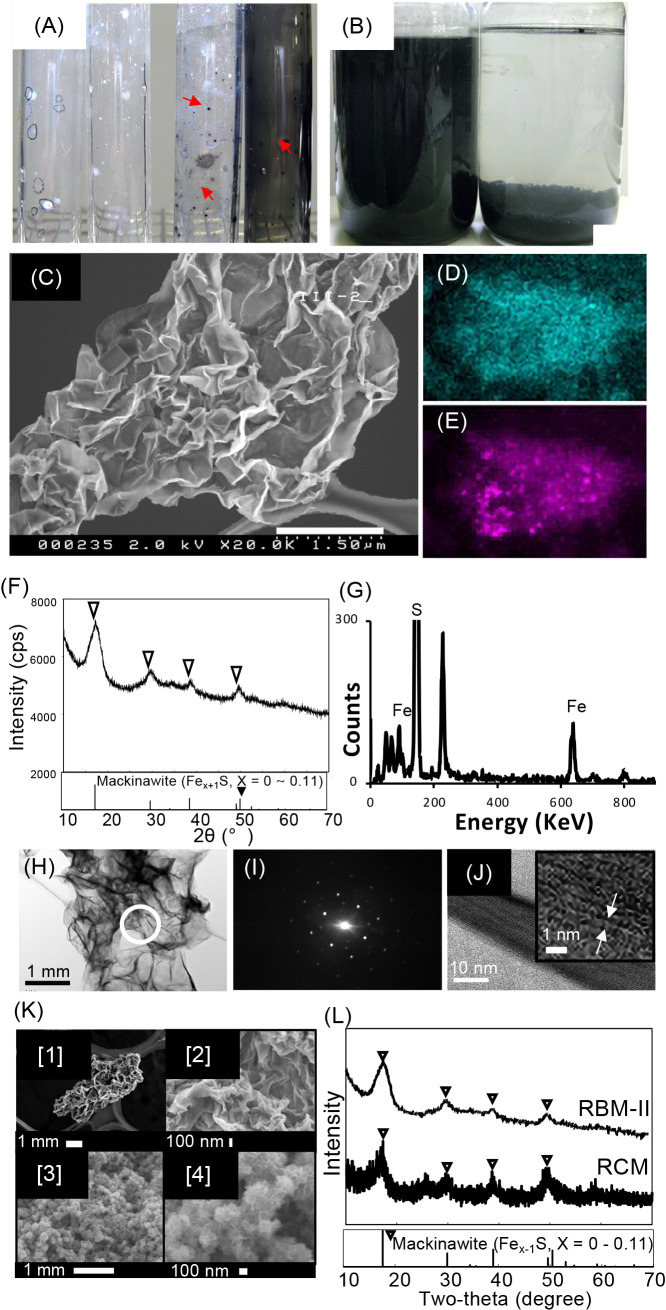
Isolation of strain HK-II and identification of the black precipitate. (A) Colonies were isolated from the anode surface of MFC using the roll tube method. Red arrows show black colonies. (B) Photographs show the black precipitate (RBM-II) produced by strain HK-II. The left bottle shows the precipitate just after agitation and the right bottle shows it after being held for 30‍ ‍min under static conditions. (C) A scanning electron microscopy (SEM) image of RBM-II. The scale bar indicates 1.50‍ ‍μm. (D) Iron mapping by an energy dispersive X-ray (EDX) ana­lysis of RBM-II, (E) sulfur mapping by the EDX ana­lysis of RBM-II, (F) the X-ray diffraction (XRD) ana­lysis profile of RBM-II, (G) the EDX ana­lysis profile of RBM-II, and (H) a transmission electron microscopy (TEM) image of RBM-II. The scale bar indicates 1‍ ‍μm. The white circle shows the area for the diffraction ana­lysis in (I), and (J) high-resolution TEM images showing mackinawite lattice fringes on RBM-II. The scale bar indicates 10‍ ‍nm. The small picture in (J) shows that the distance between layers was 5 Å, which corresponded to the reference values for pure mackinawite. (K) SEM observations of RBM-II and CSM. (K_1): RBM-II. The white scale bar indicates 1‍ ‍μm, (K_2): RBM-II. The scale bar indicates 100‍ ‍nm, (K_3): CSM. The scale bar indicates 1‍ ‍μm, (K_.4): CSM. The scale bar indicates 100‍ ‍nm. (L) XRD ana­lyses of RBM-II and CSM. The triangle indicates representative peaks of mackinawite shown in powder diffraction file #86-0389. RBM-II: rechargeable biomineral induced by strain HK-II; CSM: rechargeable chemically synthesized mackinawite.

A previous study reported that an MFC constructed with BE medium (Rubaba *et al.*, 2013), sodium lactate as an electron donor, and lake sediment as an inoculum produced a high-power density of more than 200 mW m^–2^ from days 168–197 (Suzuki *et al.*, 2018). In the present study, a part of the anode in the MFC was sampled anaerobically in a COY chamber (COY Laboratory Products) on day 205, when the power density stabilized at 5.2 mW m^–2^. Modified BE medium (BELF medium), which was supplied with 0.1‍ ‍mM Fe (III)-EDTA, was used to isolate bacteria with 0.0075% titanium (III) citrate and 20‍ ‍mM sodium lactate. White, brown, grey, and black colonies were then obtained from biofilms on the anode surface of the MFC (Suzuki *et al.*, 2018) using the roll tube method (Fig. 1A). When the grey colony was incubated in BELF medium with 0.5‍ ‍mM Fe (III) citrate instead of 0.1‍ ‍mM Fe (III)-EDTA (M-BELF medium), a black precipitate was produced; however, the color of the precipitate was initially dark white before changing to black. Therefore, we attempted to purify microorganisms from the culture using the six-well plate method (Nakamura *et al.*, 2011) with M-BELF medium. White and black colonies were obtained and purified twice using the six-well plate method. An isolated colony was inoculated into M-BELF medium, the color of the precipitate was confirmed to be black from the beginning (Fig. 1B), and microscopic observations revealed that the cell shape was homogeneous. Secondary modified BE medium (LS medium), consisting of 0.5‍ ‍g KH_2_PO_4_, 0.5‍ ‍g KH_4_Cl, 2.5‍ ‍g NaHCO_3_, 0.16‍ ‍g MgCl_2_·6H_2_O, 1.0‍ ‍mL SL8 solution, 1.0‍ ‍mL Se/W solution, 0.15‍ ‍g CaCl_2_·2H_2_O, 40‍ ‍mM sodium lactate, 20‍ ‍mM disodium sulfate, 0.5‍ ‍mg resazurin, 0.0075% titanium (III) citrate, and 1.0‍ ‍mL vitamin solution PV1 (Hiraishi *et al.*, 2002), was used to incubate the isolated strain and prevent the formation of the black precipitate. DNA was extracted and an almost full-length 16S rRNA gene was amplified using PCR with the primers 5′-AGAGTTTGATCCTG GCTCAG-3′ and 5′-AAGGAGGTGATCCAGCC-3′. The 16S rRNA gene nucleotide sequence was analyzed using a model 377 DNA sequencer (Applied Biosystems) with accession number LC612775. A GenBank database search was conducted using BLAST version 2.11.0. A neighbor-joining tree (Saitou and Nei, 1987) was constructed using njplot software in ClustalW version 1.7 (Fig. S1). These results confirmed the purity of the isolate, which was named strain HK-II.

### Electrochemical ana­lysis of the black precipitate produced by strain HK-II

Strain HK-II was incubated in M-BELF medium. The black precipitate produced was collected by filtration using a membrane filter (pore size of 0.1‍ ‍μm; Advantec) and was washed with M-BELF medium, excluding sodium lactate, MgSO_4_·7H_2_O, and Fe (III) citrate, under anaerobic conditions in a COY chamber (COY Lab.). A portion of the black precipitate collected on the filter was suspended in 2.0‍ ‍mL M-BELF medium and was attached to a carbon felt using a suction pump. The carbon felt attached to the black precipitate was set in a cell (volume of 7‍ ‍cm^3^) with a three-electrode system comprising the carbon felt as the working electrode, a platinum wire as the counter electrode, and Ag/AgCl reference electrodes (HX-R6; Hokuto Denko). The cell was then filled with M-BELF medium, excluding sodium lactate, MgSO_4_·7H_2_O, and Fe (III) citrate. Low-scan cyclic voltammetry (LSCV) was performed at a scan rate of 1 mV s^–1^ between –1.1 and 0.8‍ ‍V vs a standard hydrogen electrode (SHE). Charging was conducted using a potentiostatic operation. The potential was set at –‍0.55‍ ‍V to charge the black precipitate according to the LSCV result, which was more negative than the lowest potential of the reductive peak. Current was monitored by connecting the potentiostatic mode (HA-151B; Hokuto Denko) to a data logger (GL200A; Graphtec). Current density plateaued for at least 30‍ ‍min, indicating that charging was complete. The black precipitate was discharged and the voltage between the anode (the carbon felt attached to the black precipitate) and the cathode was monitored via 10‍ ‍Ω as external resistance. When the voltage was nearly stable (~0 V), discharge was considered to be complete. The carbon felt without the black precipitate was used as a control in CV and rechargeable ana­lyses. Capacitance was calculated using the following formulas: *V*=*IR* and *C*=*IT*, where *V* is voltage (V), *I* is current (A), *R* is resistance (Ω), *C* is Coulomb (C), and *T* is time (s). The physicochemical parameters of the single-chamber MFC were monitored during charge and discharge cycles, and pH was measured using a liquid sample with a pH meter (twinpH or B-212; Horiba). Sulfate was measured using high-performance liquid chromatography (HPLC) equipped with a Shodex IC NI-424 column (100×4.6‍ ‍mm) (Showa Denko) and a conductivity detector (Shodex CD-5; Showa Denko). The concentration of sulfide was measured using the methylene blue colorimetric method. The proportion of sulfur to iron and the percentages of oxygen and phosphorus were calculated using energy-dispersive X-ray (EDX) spectrometry. Chemically synthesized mackinawite (CSM) was analyzed in addition to rechargeable biogenic mackinawite.

### Scanning electron microscopy (SEM)

SEM observations and qualitative elemental ana­lyses of the black precipitate were performed using an SEM instrument (S-4500; Hitachi) equipped with an EDX spectrometer. The culture sample (~20‍ ‍μL) was dispersed onto a carbon-coated copper grid mounted on a standard aluminum stub. The acceleration voltage applied for EDX was 15 kV.

### EDX ana­lysis

Strain HK-II was incubated in M-BELF medium for 2‍ ‍weeks. The black precipitate was collected by filtration with a membrane filter (pore size of 0.1‍ ‍μm; Advantec) under anaerobic conditions in a COY chamber. The black precipitate on the membrane was subjected to an EDX ana­lysis (Miniscope TM3000; Hitachi). The acceleration voltage was set to 15.0 kV and the measurement time to 15 s.

### X-ray diffraction (XRD) ana­lysis

Strain HK-II was incubated in M-BELF medium for 9 days and the RBM produced was collected by filtration with a membrane filter (pore size of 0.1‍ ‍μm, Advantec) under anaerobic conditions in a COY chamber and stored under anaerobic conditions until analyzed. An XRD ana­lysis was conducted with Cu-*K*α radiation (wavelength of 1.5618 Å) at a scan rate of 5° min^–1^ from 10°–70° at 0.02° steps in 2*θ* at an output of 1.2 kW using X-ray ana­lysis instrumentation (RINT2200; Rigaku). The average diameters of the crystalline domains were assessed from XRD patterns using the Scherrer equation: *L*=*Kλ*(*β*cos*θ*)^–1^ (Wolthers *et al.*, 2003), where *L* is the average diameter of the domain, *K* is the Scherrer constant (0.91), *λ* is the wavelength of applied X-rays (1.5418 Å), *β* is the full width (in radians) at half maximum of the peak, and *θ* is the angle at the peak position.

### MFC configuration and operation

Mediator-less and air-cathode MFCs were constructed to investigate whether the black precipitate (named rechargeable biogenic mineral induced by strain HK-II [RBM-II]) was capable of charging electrons produced by microorganisms. A carbon paper electroplated with platinum (0.5‍ ‍mg cm^–2^) on one side was used as the cathode electrode (Chemix), providing a total projected cathode surface area (on one side) of 3.06‍ ‍cm^2^ (a window of 1.75‍ ‍cm set on the outside of the MFC). A proton exchange membrane (Nafion 117, Dupont) was placed between the anode and cathode. Graphite felts (SOHGOH-C) were used as the anode (4×4×0.5‍ ‍cm) and packed in the anode chamber (capacity of 50‍ ‍mL) to provide a projected anode surface area of 40‍ ‍cm^2^. RBM-II was collected on a membrane filter with a pore size of 0.1‍ ‍μm (OmniporeFM membrane filter; Merck Millipore) using a suction pump and was washed three times with autoclaved anaerobic dH_2_O. Before setting the anode in the anode chamber, a slit (~2.5×3‍ ‍cm) was made in the anode by cutting it with an autoclaved knife, and RBM-II was inserted into the slit (anode-RBM-II electrode). The slits were sewn using a fishing line. Three MFCs equipped with an RBM-II anode were constructed: RBM-MFC-1, -2, and -3, into which 0.24, 0.25, and 0.33‍ ‍g of RBM-II, respectively, was added. RBM-II was not added to control-MFC-1, -2, or -3. Strain HK-II was incubated in LS medium, and cells were collected by centrifugation (4,000×*g* for 40‍ ‍min) after sulfate was almost completely consumed by the strain HK-II sulfate-reducing process (the detection limit was 2.5‍ ‍μM). Cells were washed thrice with LS medium without sodium lactate. Cells were then resuspended in LS medium and inoculated at OD_600‍ ‍nm_ of 0.2 into all MFC anode chambers. Sodium lactate at 30‍ ‍mM was added as an electron donor. External resistance (51‍ ‍Ω) was connected between the anode and cathode using a platinum wire. All procedures were performed in a COY chamber under anaerobic conditions. All MFC voltages were recorded at 5-min intervals across a resistance of 51‍ ‍Ω using a data logger. All MFCs were run under semi-batch conditions at 25°C, and fresh sodium lactate was added at a final concentration of 30‍ ‍mM when lactate was consumed by the sulfate-reducing process of strain HK-II. Since all MFC voltages were stable after the addition of sodium lactate on day 39, the circuit was opened to charge electrons into RBM-II for 4‍ ‍h and was then closed. The data logger and external resistance were detached from MFCs under open circuit conditions. When RBM-II was charged, current density after the reclosed circuit was higher than that before the open circuit. The culture solution in the anode chamber was adequately sampled and OD_600nm_ and organic acid concentrations were measured using a spectrophotometer (UV-1800; Shimadzu) and HPLC (GL-7410 and 7450; GL Science), respectively. The charge capacity was calculated using the following formula: *C*=*IT*, where *C* is Coulomb (C), *I* is current (A), and *T* is time (s). *T* was defined as time (s) when the circuit was reclosed to the time when current density decreased to the level before the circuit was opened. Coulombic efficiency was obtained by calculating the ratio of charged capacitance to the theoretical amount of coulombs produced by organic acid consumption during the open circuit (4‍ ‍h).

### Chemically synthesized mackinawite

Mackinawite was synthesized chemically by mixing equal volumes of 100‍ ‍mM Na_2_S·9H_2_O and 100‍ ‍mM Fe(SO_4_)_2_ (NH_4_)_2_·6H_2_O under anaerobic conditions (Liu *et al.*, 2008) to obtain CSM. Before mixing these solutions, the headspace gas in a bottle containing Na_2_S·9H_2_O solution was exchanged twice with nitrogen gas for 20‍ ‍min, and the Fe(SO_4_)_2_ (NH_4_)_2_·6H_2_O solution was degassed for 60‍ ‍min and purged twice with high-purity nitrogen gas for 40‍ ‍min. CSM and RBM-II were analyzed.

### Chemical ana­lysis

Liquid samples were collected from all MFCs. They were then filtered (Millipore LG [pore size: 0.2‍ ‍μm, diameter: 13‍ ‍mm]; Merck Millipore) for the quantification of organic acids using HPLC equipped with a Shodex RSpak KC-811 column (300×8.0‍ ‍mm) (Showa Denko) and UV detector. The column heater was set to 50°C. Separation was performed using 0.1% H_3_PO_4_ solution as the mobile phase, delivered at 1.0‍ ‍mL‍ ‍min^–1^, and elution was monitored at 210‍ ‍nm. Formate, pyruvate, lactate, butyrate, and acetate were identified according to their retention times, and their concentrations were measured by comparing the peak area with that of the cognate standard sample. pH and sulfate and sulfide concentrations were measured as described above.

## Results

### Material characterization and SEM observations of the black precipitate and CSM

The isolated bacterium formed a black precipitate (Fig. 1A and B). SEM observations showed that the black precipitate was thin and frilled (Fig. 1C and K [1] and [2]). The EDX ana­lysis revealed that the black precipitate mainly consisted of iron and sulfur (Fig. 1D, E, and G). The XRD ana­lysis showed that the black precipitate was crystalline and peaked at 2 *θ*=17.61°, 30.09°, 38.99°, and 49.55° corresponding to [001], [101], [111], and [200] mackinawite, respectively (powder diffraction file #86-0389) (Fig. 1F). The XRD ana­lysis also revealed that the average crystal size of the black precipitate was estimated to be 9.2±1.5‍ ‍nm (Table S1). The diffraction pattern confirmed that the black precipitate was mackinawite (Fig. 1H and I) and a crystal layered structure was formed with a distance of 5.03 Å between the layers (Fig. 1J), which corresponded to the reference values for pure mackinawite (Csakberenyi-Malasics *et al.*, 2012). These results demonstrated that the black precipitate was biogenic mackinawite (Fe_1+_*_X_*S, *x*=0~0.11).

SEM observations showed that the CSM morphology was an aggregate of particles with diameters of 100~200‍ ‍nm with a frilled form on the particle surface (Fig. 1K.3 and 4). As expected, the XRD ana­lysis revealed that CSM was crystalline and peaked at 2 *θ*=17.61°, 30.09°, 38.99°, and 49.55° corresponding to (001), (101), (111), and (200) mackinawite, respectively (Fig. 1L) (powder diffraction file #86-0389), which corroborated CSM being mackinawite (Fe_1+x_S, x=0~0.11) as previously reported (Liu *et al.*, 2008). IN the XRD ana­lysis, the average crystal size of CSM was estimated to be 10±0.9‍ ‍nm (Table S1).

### Electrochemical and rechargeable properties of CSM and the black precipitate

**Fig. 2.**
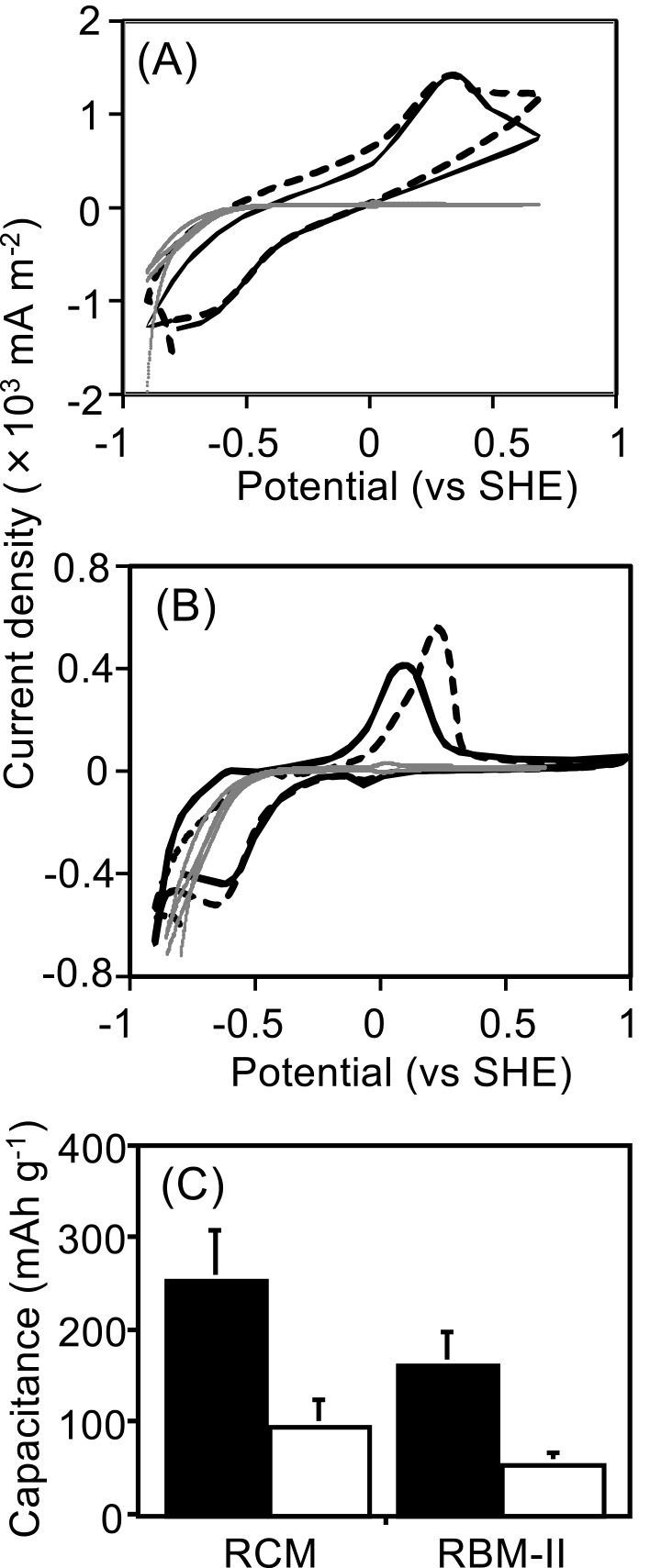
Rechargeable properties of CSM and RBM-II. (A) Cyclic voltammetry profiles of CSM. (B) Cyclic voltammetry profiles of RBM-II. The broken and solid lines show the first and second cycles, respectively. The gray lines show the cyclic voltammetry profiles of the carbon felt as the control. (C) The charge and discharge capacitances of CSM and RBM-II are shown as black and white bars, respectively. The carbon felt did not have rechargeable properties.

A cyclic voltammetry (CV) ana­lysis was conducted to electrochemically characterize CSM and the black precipitate (Fig. 2A and B). The carbon felt did not exhibit any redox peaks or capacitance. CSM showed oxidative and reductive peak potentials of 0.34‍ ‍V (vs SHE on the 1^st^ and 2^nd^ cycles) and –0.64‍ ‍V (vs SHE on the 1^st^ and 2^nd^ cycles), respectively (Fig. 2A). The black precipitate showed oxidative peak potentials of 0.24‍ ‍V (vs SHE on the 1^st^ cycle)‍ ‍and 0.17‍ ‍V (vs SHE on the 2^nd^ cycle), whereas reductive peak potentials were –0.60‍ ‍V (vs SHE on the 1^st^ cycle) and –‍0.55‍ ‍V (vs SHE on the 2^nd^ cycle) (Fig. 2B). Since these results indicated that CSM and the black precipitate had redox sites, the rechargeable properties of CSM and the black precipitate were investigated. The charge and discharge capacitances of CSM were 250±57 and 94±30‍ ‍mAh‍ ‍g^–1^, respectively, whereas those of the black precipitate were 160±35 and 53±14 mAh g^–1^, respectively (Fig. 2C). The black precipitate was named RBM-II. RBM-II exhibited discharge and charge properties repeatedly after three discharge and charge cycles, demonstrating the rechargeability of RBM-II (Supplemental Fig. S6).

### pH and concentrations of sulfide and elements in CSM and RBM-II under charged and discharged conditions

**Fig. 3.**
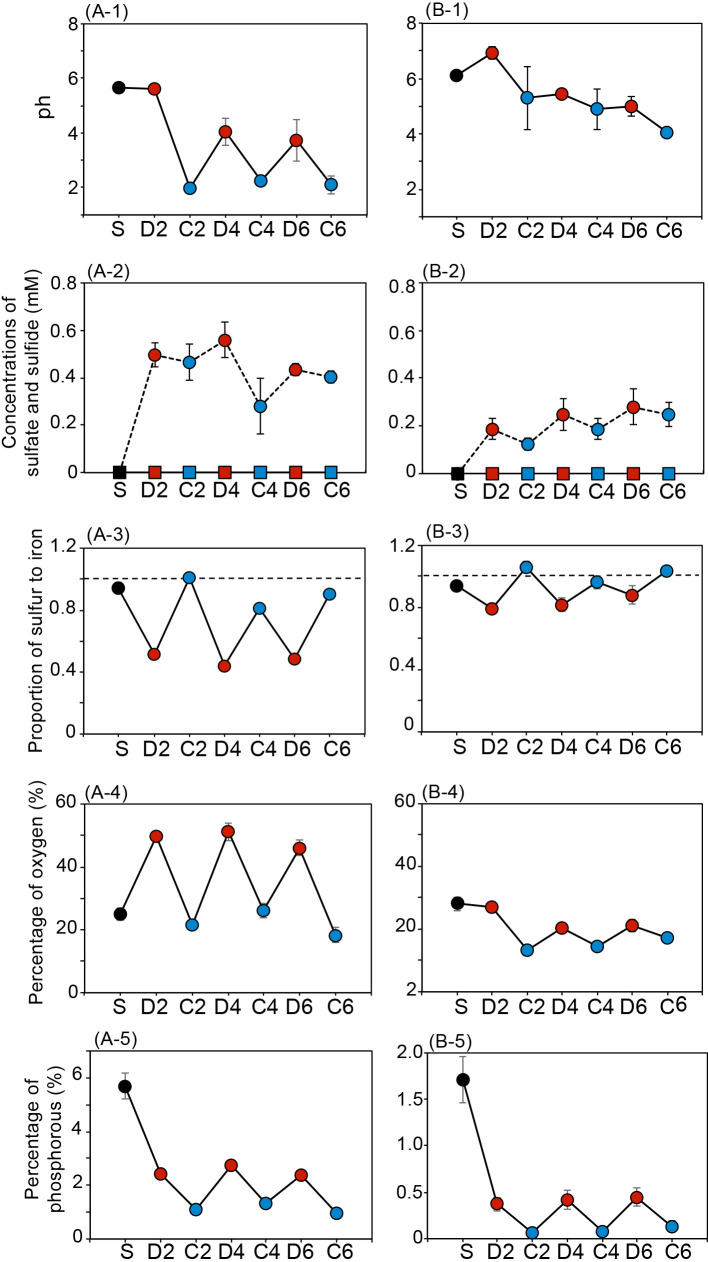
Physicochemical parameters of CSM and RBM-II under charged and discharged conditions. (A-1_5) CSM; (B-1_5) RBM-II; (A-1) and (B-1) pH; (A-2) and (B-2) concentrations of sulfide (broken line) and sulfate (solid line); (A-3) and (B-3) the proportion of sulfur to iron. The broken line denotes that the proportion of sulfur to iron was 1.0; (A-4) and (B-4) the percentage of oxygen; (A-5) and (B-5) the percentage of phosphorous. S: Initial CSM samples synthesized by chemical reactions or RBM-II induced by strain HK-II. The CSM and RBM-II samples were initially charged. D2: Discharged samples prepared with one discharge and charge cycle and a 2^nd^ discharge cycle. C2: Charged samples prepared with two discharge and charge cycles. D4: Discharged samples prepared with three discharge and charge cycles and a 4^th^ discharge cycle. C4: Charged samples prepared with four discharge and charge cycles. D6: Discharged samples prepared with five discharge and charge cycles and a 6^th^ discharge cycle. C6: Charged samples prepared with six discharge and charge cycles.

**Fig. 4.**
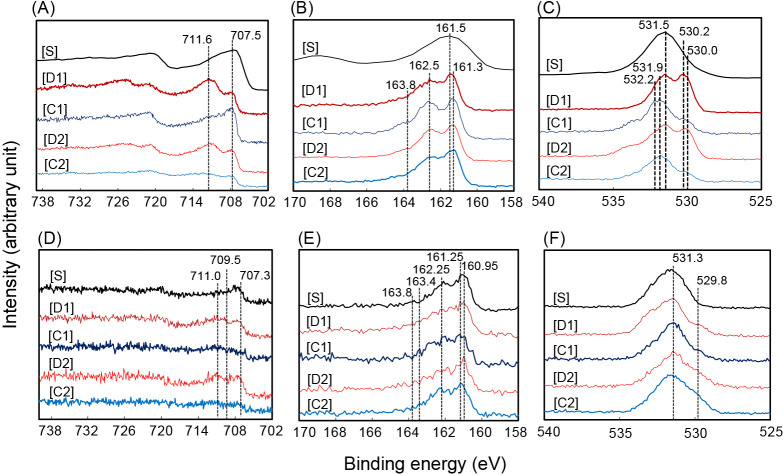
Narrow scans of XPS ana­lyses of CSM and RBM-II under discharge and charge conditions. (A) Fe(2p^3/2^), (B) S(2p), and (C) O(1s) of CSM, respectively. (D) Fe(2p^3/2^), (E) S(2p), and (F) O(1s) of RBM-II, respectively. [S]: The initial sample when CSM was synthesized chemically or after RMB-II was produced by strain HK-II; [D]: discharged sample; [C]: charged sample, the numbers beside “D” and “C” denote the numbers of discharge and charge cycles. Binding energies for Fe(2p^3/2^), S(2p), and O(1s) peaks are described in the figure with dashed lines and are shown in supplemental table S2.

**Fig. 5.**
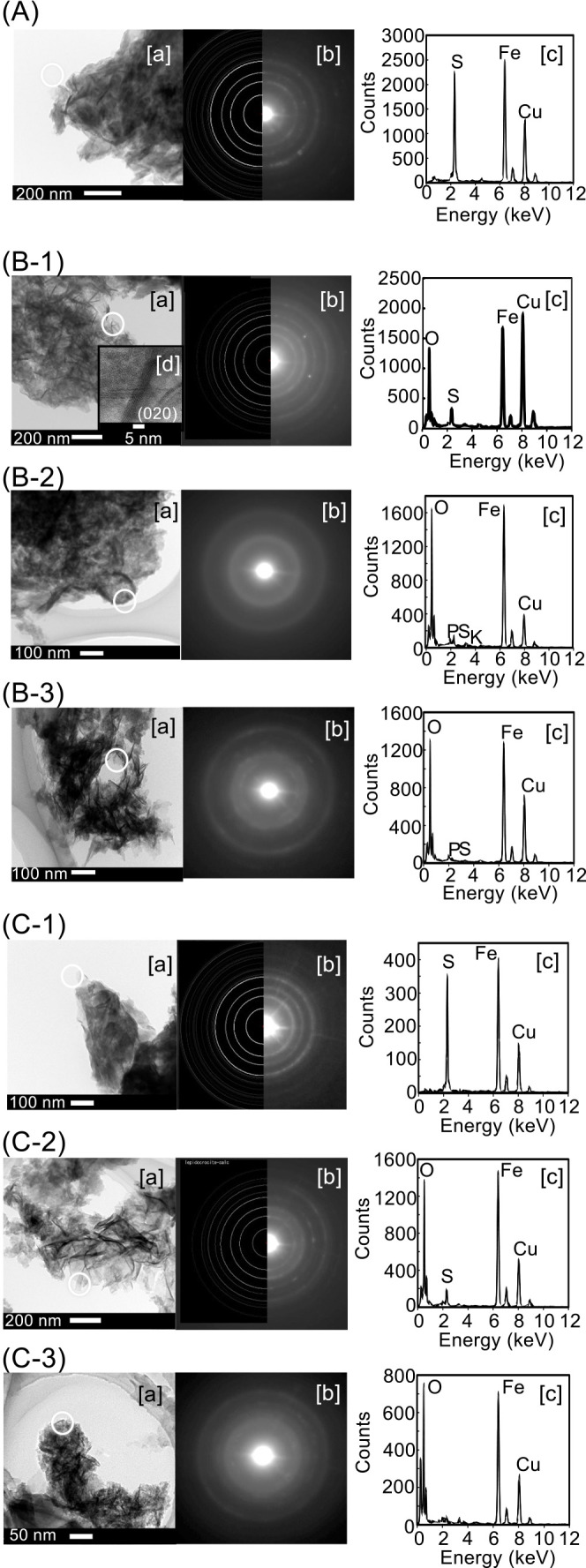
Form changes of RBM-II confirmed by FE-TEM. (A) RBM induced by strain HK-II. (B) The 2^nd^ discharged RBM-II was analyzed at three different positions (B-1, B-2, and B-3). (C) The 2^nd^ charged RBM-II was analyzed at three different positions (C-1, C-2, and C-3). [a] High-resolution TEM images, [b] electron diffraction patterns at a selected area, and [c] EDX spectra, [d] in B-1-[a]: Displaying characteristic layers of lepidocrocite (020). The white bar presents a size marker. A white circle shows the area for the diffraction ana­lysis.

The pH of the CSM suspension decreased from 5.6 under 2^nd^ discharged conditions to 1.8±0.20 under 2^nd^ charged conditions. pH in the suspension then regularly changed from 4.0±1.5 under discharged conditions to 2.6±1.1 under charged conditions (Fig. 3A-1). The concentration of sulfide in the CSM suspension increased by 0.50±0.051‍ ‍μM under 2^nd^ discharged conditions and decreased by 0.47±0.076‍ ‍μM under 2^nd^ charged conditions (Fig. 3A-2), after which it slightly increased and decreased under discharged and charged conditions, respectively. Furthermore, sulfate was not detected in the CSM suspension during the experiment. The proportion of sulfur to iron changed periodically corresponding to charged and discharged conditions, which were 0.48±0.036 under discharged conditions and 0.91±0.10 under charged conditions (Fig. 3A-3). Moreover, the percentages of oxygen and phosphorus changed periodically, corresponding to charged and discharged conditions. The percentage of oxygen in CSM was 49±2.6% under discharged conditions and 22±4.0% under charged conditions (Fig. 3A-4), whereas the percentage of phosphorus in CSM was 2.5±0.17% under discharged conditions and 1.1±0.18% under charged conditions (Fig. 3A-5).

Conversely, the pH of the RBM-II suspension decreased from 6.9±0.22 under 2^nd^ discharged conditions to 5.3±1.1 under 2^nd^ charged conditions (Fig. 3B-1). A reduction in the pH of the suspension was not observed under discharged conditions, whereas a decrease was noted under charged conditions. Sulfate was not detected in the RBM-II suspension (Fig. 3B-2), whereas the concentration of sulfide in the RBM-II suspension increased to 0.18±0.044‍ ‍μM under 2^nd^ discharged conditions and decreased to 0.12±0.025‍ ‍μM under 2^nd^ charged conditions. The concentration of sulfide increased and decreased slightly under 4^th^ and 6^th^ discharged and charged conditions, respectively (Fig. 3B-2). The proportion of sulfur to iron changed periodically, corresponding to charged and discharged conditions. The proportion of sulfur to iron was 0.83±0.046 under discharged conditions and 1.0±0.053 under charged conditions (Fig. 3B-3). The percentages of oxygen and phosphorus changed periodically corresponding to charged and discharged conditions. The percentage of oxygen in RBM-II was 23±3.7% under discharged conditions and 15±2.1% under charged conditions (Fig. 3B-4), whereas the percentage of phosphorus in RBM-II was 0.42±0.042% under discharged conditions and 0.083%±0.037% under charged conditions (Fig. 3B-5).

### Form changes in CSM and RBM-II

An XPS ana­lysis was performed to confirm whether CSM and RBM-II changed in accordance with charged and discharged conditions. In CSM, the binding energy of Fe(2P^3/2^) at 711.6 eV corresponding to the Fe(III)-O band (McIntyre and Zetaruk, 1977) was higher than those of 707.3 and 707.5 eV corresponding to the Fe(II)-S bond (Jones *et al.*, 1992; Herbert *et al.*, 1998) under discharged conditions (Fig. 4A-[D1] and [D2]) and vice versa (Fig. 4A-[S], [C1], and [C2]). In RBM-II, the binding energy of Fe(2P^3/2^) at 711.0 eV corresponding to Fe(III)-O (Thomas *et al.*, 1998) was higher than that of 707.3 eV corresponding to the Fe(II)-S bond (Jones *et al.*, 1992) under discharged conditions (Fig. 4D-[D1] and [D2]) and vice versa (Fig. 4D-[S], and [C2]). The binding energy of Fe(2P^3/2^) at 709.5 eV corresponding to Fe(II)-O (McIntyre and Zetaruk, 1977) increased and decreased after discharged and charged treatments, respectively (Fig. 4D).

One broad peak corresponding to the binding energy of S(2p) at approximately 161.5 eV was observed in the initial CSM (Fig. 4B-[S]), whereas the two main peaks at 161.3 and 162.5 eV corresponding to monosulfide (Pratt *et al.*, 1994) and disulfide (Mycroft *et al.*, 1990), respectively, were always observed under discharged and charged conditions (Fig. 4B). In RBM-II and CSM, the two main peaks of the binding energy of S(2p) at 160.95 and 162.25 eV corresponding to monosulfide (Herbert *et al.*, 1998) and disulfide (Pratt *et al.*, 1994), respectively, were observed under all conditions, with the exception of 1^st^ discharged conditions (Fig. 4E).

The binding energy of O(1s) at 531.5 eV corresponding to the OH^–^ component (Herbert *et al.*, 1998) was observed in the initial CSM and was one of the main contributors to discharged conditions (Fig. 4C-[S], [D1], and [D2]). A new broad peak in the binding energy of O(1s) at 530.0 eV (Mycroft *et al.*, 1990) and 530.2 eV (McIntyre and Zetaruk, 1977) corresponding to O^2–^ was observed under discharged conditions (Fig. 4C-[D1] and [D2]). The broad peak disappeared after charging and only one broad peak of binding energy at approximately 531.9 and 532.2 eV was observed, but not identified (Fig. 4C-[C1] and [C2]). In RBM-II, the binding energy of O(1s) at 531.3 eV corresponding to the OH^–^ component (Mullet *et al.*, 2002) was a major contributor in RBM-II under all conditions (Fig. 4F). The binding energies of the Fe(2P3/2), S(2p), and O(1s) peaks are listed in Table S2.

FE-TEM ana­lyses were performed to investigate the form change details of RBM-II. TEM observations showed that RBM-II was in the form of a film (Fig. 5A[a]). Electron diffraction patterns and EDX ana­lyses revealed that the charged RBM-II was mackinawite (Fig. 5A[b] and -[c]). After the 2^nd^ discharge, a scaly form was observed (Fig. 5B[a]), and the presence of lepidocrocite was confirmed in the selected area using electron diffraction patterns and EDX ana­lyses (Fig. 5B-1-[b] and -[c]). High-resolution TEM revealed a nanocrystal lepidocrocite consisting of a (020) plane with spacing of 0.64 Å (Fig. 5B-1-[d]), and amorphous oxidized irons were also observed (Fig. 5B-2 and 5B-3). After the 2^nd^ charge, the electron diffraction pattern and EDX ana­lyses demonstrated that RBM-II consisted of mackinawite (Fig. 5C-1), whereas lepidocrocite and amorphous iron oxide were observed in another selected area (Fig. 5C-2 and 5C-3, respectively). These results indicate that RBM-II consisted of mackinawite, lepidocrocite, and amorphous iron oxide after the 2^nd^ discharge treatment.

### Improvement of MFCs using RBM-II

**Fig. 6.**
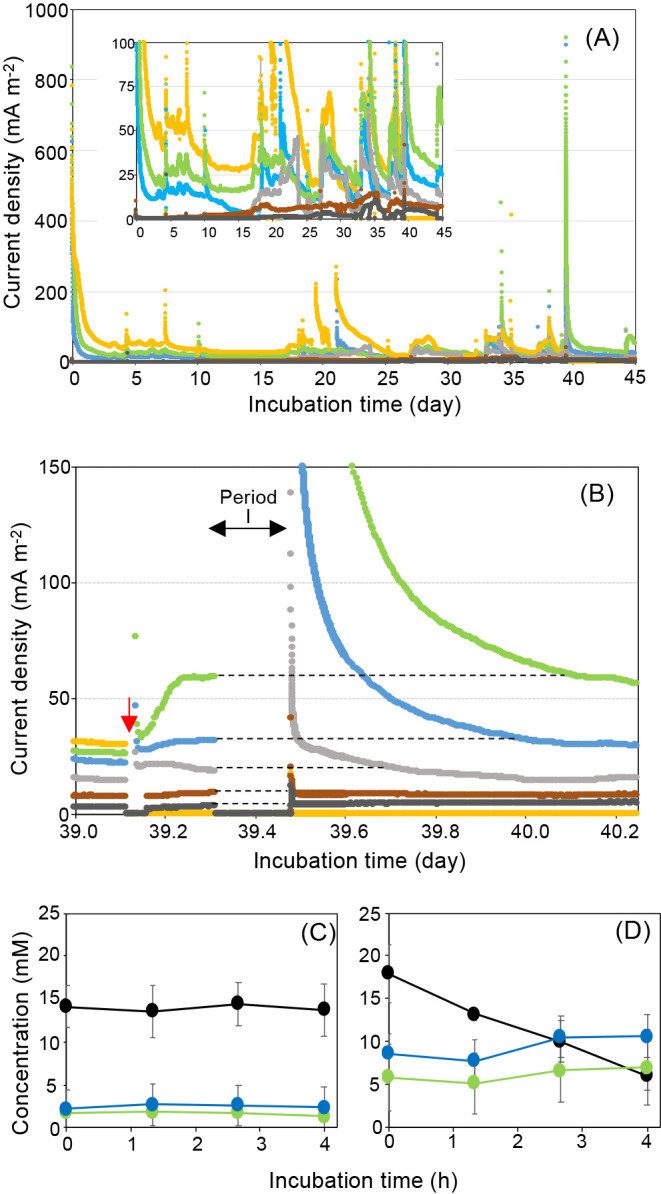
Current production by MFCs equipped with RBM-II. (A) and (B) Current densities of control MFCs and RBM-MFCs. Gray, brown, and black lines show the current densities of control MFC1, 2, and 3, respectively. Blue, green, and orange lines show the current densities of RBM-MFC1, 2, and 3, respectively. The small figure in figure A shows the difference in current density between control MFCs and RBM-MFCs. (B) Current densities of all MFCs under closed- and open-circuit conditions. The red arrow shows the time of lactate addition to all MFCs. Period I shows the time under open-circuit conditions for all MFCs. The data logger was removed from the MFCs during period I so that current densities were zero. The electrode wire of RBM-MFC3 was broken when the anode and cathode were connected after sampling. Therefore, the current from RBM-MFC3 was not detected (orange line). (C) Concentrations of organic acids during the open circuit (Period I) in control MFCs. Black circles, lactate; blue circles, acetate; green circles, propionate. (D) Concentrations of organic acids during the open circuit (Period I) in RBM-MFCs. Black circles, lactate; blue circles, acetate; green circles: propionate.

The average current densities of control-MFCs and RBM-MFCs were 5.8±2.8 and 18±9.9 mA m^–2^, respectively, during initial lactate consumption, after which the lactate consumption rate and current density increased and were stable in all MFCs (Fig. 6A and S4). Current density became stable at 31±2.7 mA m^–2^ in RBM-MFC1 and 58±2.1 mA m^–2^ in RBM-MFC2 after the addition of fresh lactate on day 39, and the circuit was then opened for 4‍ ‍h (Period I shown in Fig. 6B) to investigate whether RBM-II was recharged by bacterial activity (Fig. 6B). Unfortunately, RBM-MFC3 was broken; therefore, its current was not measurable during the experiments. After the circuit re-closed, the currents produced from RMB-MFC1 and -MFC2 were 120±90 and 250±140 mA m^–2^, respectively (Fig. 6B). Higher current production was maintained for 13.6‍ ‍h and 22.9‍ ‍h in RBM-MFC1 and -MFC2, respectively (Fig. 6B). During Period I, the concentrations of lactate, acetate, and propionate were stable in all control-MFCs (Fig. 6C), whereas the concentration of lactate decreased and those of acetate and propionate increased in all RBM-MFCs (Fig. 6D). Coulombs produced from consumed organic acids were estimated to be approximately 1,060 and 450 kC in RBM-MFC1 and -MFC2, respectively. The discharged capacitances of RBM-MFC1 and -MFC2 practically were 170 and 390 kC, respectively (Fig. 6B). Coulombic efficiencies for charged capacitance were 16 and 86% in RBM-MFC1 and -MFC2, respectively.

## Discussion

The present study demonstrated that RBM-II, a biogenic mackinawite induced by *Nitratidesulfovibrio* sp. strain HK-II under sulfate-reducing conditions with ferric iron, possessed rechargeable properties for at least three discharge and charge cycles (Fig. S6). To the best of our knowledge, this is the first study to report this result; however, mackinawite is known to be a conductive mineral (Nakamura *et al.*, 2009). Magnetite (Fe[II]Fe[III]_2_O_4_) is a naturally occurring rechargeable mineral in which the redox cycling of Fe(II) and Fe(III) occurs via a rechargeable mechanism (Byrne *et al.*, 2015). Conversely, as a rechargeable mechanism of RBM-II, according to regular changes in physiochemical properties under charge and discharge cycles (Fig. 3, 4, and 5), the following simple reactions were predicted (Fig. S5-A) when the form of RBM-II was completely changed under discharged and charged conditions. When considering the discharge reaction from mackinawite to lepidocrocite, 4*FeS*+8*H*_2_*O* → 4γ-*FeOOH*+4*H*_2_*S*+4*H*^+^+4*e*^–^ (the reaction on anode [1]) and 4*H*^+^+*O*_2_+4*e*^–^ → 2*H*_2_*O* (the reaction on cathode [2]), the complete reaction is as follows: 4*FeS*+6*H*_2_*O*+*O*_2_ → 4γ-*FeOOH*+4*H*_2_*S* (total reaction [3]). The charge reaction from lepidocrocite to mackinawite is as follows: 4γ-*FeOOH*+4*H*_2_*S*+8*e*^–^ → 4*FeS*+2*O*_2_+4*H*_2_*O*+4*H*^+^ (reaction [4]) (Fig. S5-B). Fe(II) was related to the discharge process in RBM-II and magnetite (Byrne *et al.*, 2015), whereas Fe(III) and H_2_S were both related to the charge process in RBM-II. These predicted reactions ([1] and [4]) suggest that the charge capacitance was 2-fold higher than the discharge capacitance in RBM-II, which is supported by the results on actual charge and discharge capacitances (Fig. 2C). The protons produced by the discharge treatment (reaction [1]) were consumed by the cathode reaction [2], indicating that pH did not decrease under discharged conditions and *vice versa* (Fig. 3B-1). Actual reactions were more complex than originally predicted because mackinawite and lepidocrocite were both present simultaneously with an increase in the number of charge and discharge cycles in RBM-II (Fig. 5C). Furthermore, amorphous iron oxide compound(s), which are mackinawite and lepidocrocite precursors (Rickard, 1969; Sweeney and Kaplan, 1973), were detected in RBM-II under charged and discharged conditions (Fig. 5B and C). Not all of the sulfide liberated under discharged conditions was used to form mackinawite from lepidocrocite because of diffusion (Fig. 3B-2), which resulted in a mixture of mackinawite, lepidocrocite, and amorphous iron oxides. A previous study reported that the formation of FeS formation may be directly linked to ferric(hydro)oxide sulfidation (Hellige *et al.*, 2012). Sulfide oxidation and the subsequent formation of polysulfides and disulfides are key reactions that produce ferrous sulfide minerals via interactions between sulfides and ferric(hydro)oxides (Peiffer *et al.*, 1992; Hellige *et al.*, 2012; Wan *et al.*, 2014). Previous studies suggested the importance of sulfur speciation in form changes (Lennie *et al.*, 1997; Burton *et al.*, 2006; Burton *et al.*, 2009). Biogenic ferrihydrite compounds are more stable than chemically synthesized compounds because of the restricted crystal growth caused by binding with bacterial cells, extracellular polysaccharides, and silica (Banfield *et al.*, 2000; Kennedy *et al.*, 2004; Najem *et al.*, 2016). Sheets of Fe atoms on the [001] plane of mackinawite are separated by *ca*. 0.5‍ ‍nm and are weakly held by van der Waals bonds between S atoms (Wolthers *et al.*, 2003), which are capable of binding cations and result in disordered mackinawite with different properties (Wolthers *et al.*, 2003; Zavasnik *et al.*, 2014). Disordered mackinawite was shown to be produced in media containing a sulfate-reducing bacterium (Herbert *et al.*, 1998). Although the average crystallite sizes of CSM and RBM-II were similar, their structures were not the same because of the differences in [101] (Table S1). These results suggest that differences in the stabilities of RBM-II and CSM are dependent on the formation process. The form change in CSM was more pronounced than that in RBM-II (Fig. 3), suggesting that the latter is more suitable for biotechnological applications.

As shown in Fig. 6A, the current densities of all RBM-MFCs were higher than those of control MFCs, indicating that RBM-II maintained stability and performance even in the air-cathode MFCs used in the present study. RBM-MFCs degraded lactate, even under open-circuit conditions, and produced high current densities after closed-circuit conditions (Fig. 6B and C), demonstrating that RBM-II is a useful rechargeable material because it charges electrons at an extremely low current density produced by bacterial cells. Electrically conductive nanoparticles have been widely used to improve current density in MFCs (Zhang *et al.*, 2013; Kaur *et al.*, 2020; Deng *et al.*, 2022); however, this is the first study to report a secondary rechargeable MFC using a biogenic mineral equipped in the anode electrodes. The coulombic efficiencies of RBM-MFCs differed (Fig. 6), suggesting that RBM-II equipped in the anode electrodes was not in sufficient contact with the anode surface. The assembly of the anode and RBM-II is an important factor affecting their practical application.

Electroactive humus and mineral particles are present in soil and sediment environments, and electroactive bacteria, including sulfate and iron reducers, produce ATP coupled with EET and EEU (Deng and Okamoto, 2018; Deng *et al.*, 2022), suggesting that electroactive microbes are relevant to geochemical reactions beyond our consideration. Deep-sea hydrothermal vents, which are environments isolated from solar energy, provide an ideal habitat for chemolithotrophic microbial communities by continuously supplying reductive energy and currents via chimneys (Nakagawa and Takai, 2008; Nakamura *et al.*, 2010; Yamamoto *et al.*, 2017), such as conductive materials and metals (Nakamura *et al.*, 2010), resulting in spontaneous and widespread electricity generation around deep-sea hydrothermal fields (Yamamoto *et al.*, 2017). Conductive consortia, microbial communities, and populations are formed around conductive minerals (Kato *et al.*, 2010; Kato *et al.*, 2013) or via direct interspecies electron transfer (DIET) (Summers *et al.*, 2010; Pfeffer *et al.*, 2012; Wegener *et al.*, 2015). Several bacteria are capable of electrosynthesis by harvesting electrons from conductive materials outside their cells (Nakamura *et al.*, 2009). The sulfate-reducing bacterium *Desulfovibrio* sp. strain JY formed conductive methanogenic aggregates with *Methanobacterium* sp. strain YSL via DIET (Nevin *et al.*, 2011).* S. loihica* strain PV-4 has been shown to self-organize an electrically conductive network using iron sulfide produced by itself, resulting in efficient metabolized electron transfer (Nakamura *et al.*, 2010; Kondo *et al.*, 2015; Zheng *et al.*, 2021). These findings suggest that biogenic rechargeable minerals, such as RBM-II, function as an electron pool for electron donors and acceptors in microbial ecosystems, which provides insights into the mechanisms by which slow-growing microbial communities survive in deeply buried sediments where obtaining cellular maintenance energy is considered to be difficult (Inagaki *et al.*, 2015; Lever *et al.*, 2015; Moller *et al.*, 2018). This expands our understanding of the mechanisms by which the ubiquity of microbial life is maintained, even in deep subseafloor environments (Kallmeyer *et al.*, 2012; Parkes *et al.*, 2014; Inagaki *et al.*, 2015) irrespective of the isolation of solar energy.

In conclusion, *Nitratidesulfovibrio* sp. strain HK-II induced a rechargeable biogenic mineral, RBM-II, which showed an incomplete reversible form change between mackinawite under charged conditions, lepidocrocite under discharged conditions, and amorphous iron oxides under both conditions. RBM-II rechargeable reactions were proposed from the results of electrochemical and material science ana­lyses, whereas CSM rechargeable reactions remain unclear. When RBM-II is used practically as a rechargeable material, its stability and safety must be confirmed through 1,000–2,000 charge and discharge cycles. In addition, material scientific modifications are required for the practical use of RBM-II. Mackinawite is a ubiquitous mineral in anaerobic environments (Rickard and Luther III, 2007), suggesting that biogenic mackinawite and CSM play roles as electron donors/acceptors in microbial ecosystems, not only in locations separated from a solar energy system, but also in ordinary anaerobic environments, such as paddy fields. The electrical energy acquisition mechanisms used by microbes via rechargeable/conductive minerals, such as RBM-II, may be a major survival strategy because biogenic minerals, such as RBM-II, play a role in electron pools as electron donors/acceptors for anaerobes. Further studies are needed to obtain a more detailed understanding of overlooked microbial functions and ecological roles via electric flow among organic compounds, minerals, and microbes.

## Citation

Arashi, Y., Mochihara, H., Kubota, H., Suzuki, K., Chiba, Y., Kato, Y., et al. (2025) A Rechargeable Biomineral Induced by the Sulfate-reducing Bacterium *Nitratidesulfovibrio* sp. HK-II. *Microbes Environ ***40**: ME24022.

https://doi.org/10.1264/jsme2.ME24022

## Supplementary Material

Supplementary Material
