# Supplementary material for: A Rechargeable Biomineral Induced by the Sulfate-reducing Bacterium Nitratidesulfovibrio sp. HK-II
Source: Microbes Environ. 2025 Jul 3;40(3):ME24022. doi: 10.1264/jsme2.ME24022 (PMC12501878; doi:10.1264/jsme2.ME24022)
Supplement: Supplementary file 1 — Supplementary Material [file 40_24022_s1.pdf]

Supplementary Figure S1

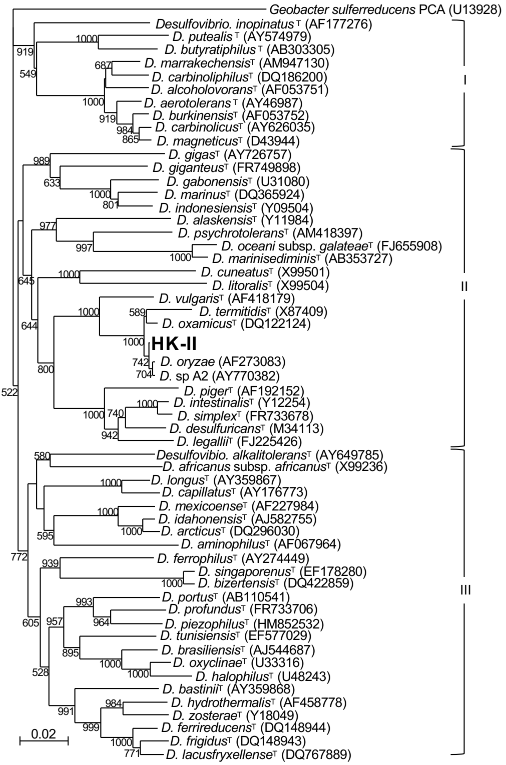

Phylogenetic analyses of the strain HK-II isolated in this study.
By homology search of the 16S rRNA gene nucleotide sequence of the strain HK-II, it was shown that the strain HK-II was closely related to *Desulfovibiro oryzae* (99.7% identity, accession number AF273083), identifying the strain HK-II phylogenetically as *Desulfovibiro* sp. strain HK-II. *Desulfovibrio* strains were grouped into three clusters, and the strain HK-II belonged to the cluster II.

Supplementary Figure S2

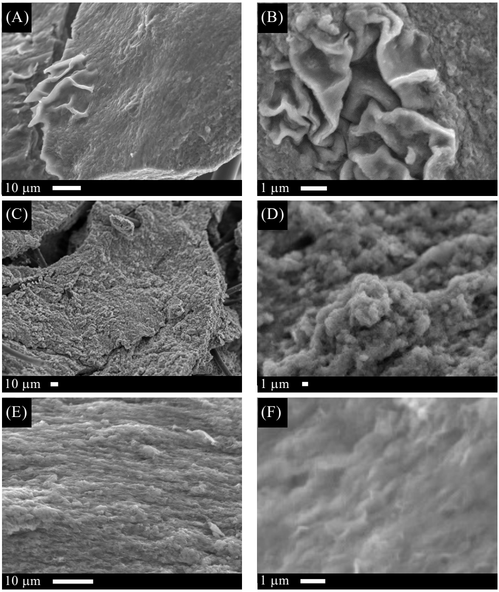

SEM observation of RBM-II on rechargeable treatments.

(A and B) RBM-II produced by strain HK-II. Since it is charged, RBM-II is mackinawite. (C and D) RBM-II after the 2nd discharge treatment. Since it is discharged, RBM-II is lepidocrocite. (E and F) RBM-II after the 2nd charge treatment. Since it is charged, RBM-II is mackinawite. White bars are size markers (10 μm or 1 μm).

Supplementary Figure S3

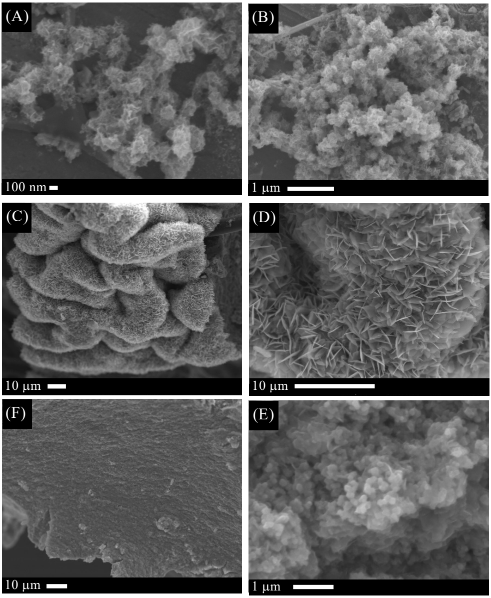

SEM observation of CSM on rechargeable treatments.
(A and B) CSM produced by chemical reactions. Since it is charged, CSM is mackinawite. (C and D) CSM after the 2nd discharge treatment. Since it is discharged, CSM is lepidocrocite. (E and F) CSM after the 2nd charge treatment. Since it is charged, CSM is mackinawite. White bars are size markers (10 μm or 1 μm).

Supplementary Figure S4

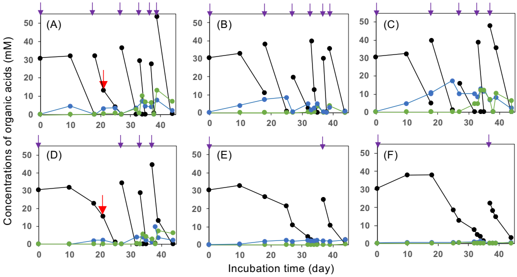

Concentrations of organic acids in MFCs.

(A-C): RBM-MFCs. (A); RBM-MFC1, (B); RBM-MFC2, (C); RBM-MFC3, (D-F): Control-MFCs. (D); control-MFC1, (E); control-MFC2, (F); control-MFC3. Black circles; lactate, blue circulars; acetate, green circulars; propionate. Red arrows donate the time of addition of sulfate. Purple arrows donate the time of addition of sodium lactate.

Supplementary Figure S5

Deduced rechargeable reactions of RBM-II

These reactions are deduced on the assumption that RBM-II is completely altered the form under the discharge and charge conditions: makinawite on charge and lepidocrocite on discharge.

A) Discharge reaction

$4FeS + 8H_2O \rightarrow 4\gamma\text{-}FeOOH + 4H_2S + 4H^+ + 4e^-$ (reaction on the anode [1])

$4H^+ + O_2 + 4e^- \rightarrow 2H_2O$ (reaction on the cathode [2])

total reaction was as follows;

$4FeS + 6H_2O + O_2 \rightarrow 4\gamma\text{-}FeOOH + 4H_2S$ (total reaction [3]).

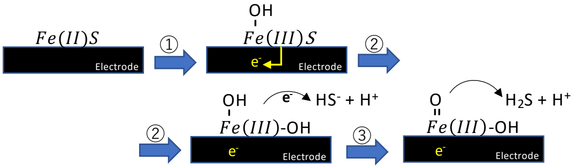

Discharge reaction on the anode was deduced as described above.
The reaction consists of 3 steps at least from ① to ③.

B) Charge reaction $4\gamma\text{-}FeOOH + 4H_2S + 8e^- \rightarrow 4FeS + 2O_2 + 4H_2O + 4H^+$

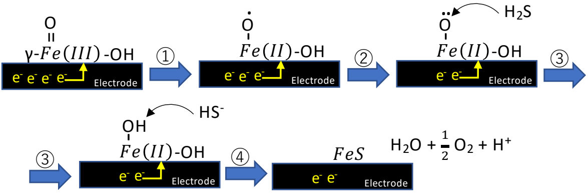

Charge reaction was deduced as described above. The reaction consists of 4 steps at least from ① to ④.

Supplementary Figure S6

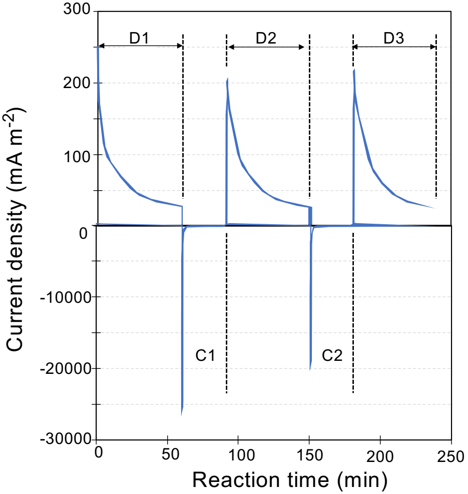

Discharge and charge treatments for RBM-II.

Strain HK-II was incubated under sulfate-reducing conditions with ferric citrate. RBM-II was induced by strain HK-II and was then collected at day. The culture was mixed well and RBM-II in 10 mL was trapped ion anode electrode. The anode electrode was set in an electrochemical cell with three electrodes. External resistance was 5100Ω.

(D1): Since RBM-II induced by strain HK-II was charged, the RBM-II was discharged.
(C1): 1st charge for 30 min at −0.55 V, (D2): 2nd discharge,
(C2): 2nd charge for 30 min at −0.55 V, (D3): 3rd discharge.

These results demonstrated that RBM-ii was rechargeable materials in 3 cycles of discharge and charge treatments at least.

Table S1. Crystallite size of RBM-II, and CSM

Plate	Two-theta [°]	crystallite size [nm]	
		RBM-II	CSM
001	17.6	6.2	8.0
101	30.1	6.3	11.5
111	39.0	12.0	12.5
200	49.5	12.3	9.6
Average		9.2±1.5	10±0.9

Table S2. Bindig energies for Fe ($2p^{3/2}$), S(2p), and O(1s) peaks in comparison with fitted peaks in Fig 4.

Binding energy (ev)		Species	References
Fe ($2p^{3/2}$)	707.1	Fe(II)-S	Pratt et al., 1994 (39)
	707.3	Fe(II)-S	Herbert et al. 1998 (36)
	707.5	Fe(II)-S	Jones et al., 1992 (37)
	709.0	Fe(II)-O	Thomas et al., 1998 (38)
	709.5	Fe(II)-O	McIntyre and Zetaruk 1977 (35)
	709.7	Fe(III)-S	Thomas et al., 1998 (38)
	711.0	Fe(III)-O	Thomas et al., 1998 (38)
	711.6	Fe(III)-O	Mullet et al., 2002 (42)
	712.0	Fe(III)-O	Thomas et al., 1998 (38)
	714.0	Fe(III)-O	Thomas et al., 1998; Pratt et al., 1994 (38, 39)
S(2p)	160.95	S^{2-}	Herbert et al., 1998 (36)
	161.25	S^{2-}	Pratt et al., 1994 (39)
	161.3	S^{2-}	Pratt et al., 1994, Mullet et al., 2002 (39, 42)
	161.4	S^{2-}	Thomas et al., 1998 (38)
	162.2	S_2^{2-}	Herbert et al. 1998 (36)
	162.25	S_2^{2-}	Herbert et al. 1998 (36)
	162.5	S_2^{2-}	Mycroft et al., 1990 (40)
	163.15	S_n^{2-}	Herbert et al. 1998 (36)
	163.4	S_n^{2-}	Thomas et al., 1998 (38)
	164.0	S_8	Thomas et al., 1998 (38)
O(1s)	529.5	O^{2-}	Mullet et al., 2002 (42)
	529.8	O^{2-}	Jones et al., 1992 (37)
	530.0	O^{2-}	Ferris et al., 1989 (41)
	530.2	O (Lepidocrocite)	McIntyre and Zetaruk 1977 (35)
	531.3	OH^-	Mullet et al., 2002 (42)
	531.4	OH^- (Lepidocrocite)	McIntyre and Zetaruk 1977 (35)
	531.5	OH^-	Herbert Jr. et al. 1998 (36)
	532.52	adsorbed H_2O	Herbert Jr. et al. 1998 (36)
	532.6	adsorbed H_2O	Pratt et al., 1994 (39)
	533.58	other H_2O	Herbert Jr. et al. 1998 (36)
